# Recent Advances in Prevention and Control of Rabies

**DOI:** 10.4061/2011/956428

**Published:** 2011-09-26

**Authors:** S. N. Madhusudana, Deborah Briggs, Hervé Bourhy

**Affiliations:** ^1^WHO Collaborating Centre for Reference and Research on Rabies and Department of Neurovirology, National Institute of Mental Health and Neurosciences, Bangalore 560029, India; ^2^Global Alliance for Rabies Control, Kansas State University, Manhattan, KS 66502, USA; ^3^Unit Lyssavirus Dynamics and Host Adaptation and WHO Collaborating Centre for Reference and Research on Rabies, Institut Pasteur, 75724 Paris Cedex 15, France

Rabies is one of the oldest zoonotic disease which continues to pose a significant threat to humans in most parts of the world, particularly in Asia and Africa. As huge animal reservoirs exist in most parts of the world, the threat to humans is likely to continue for many more years. In the past decade, a renewed effort has been made to reduce the burden of rabies focusing particularly on the Asian and African countries. Several national and international organizations including World Health Organization (WHO) and Global Alliance for rabies Control (GARC) are now actively working out various strategies for getting international and national commitment to eliminate human rabies and reduce canine rabies. New developments have taken place in rabies prevention in humans and canine rabies control. Keeping this view, a special issue of Journal of Preventive Medicine was designed to update the readers on important aspects of rabies prevention, epidemiology and control. There are six well written and informative papers in this issue. The first paper by G. Gongal and A. E. Wright deals with the current rabies scenario in the world with special reference to south east Asian countries which contribute nearly 60% of global burden. The paper describes the current trends in human and animal rabies in this region and newly initiated preventive and control efforts to eliminate dog-mediated human rabies in this region. The second paper by B. Dodet et al. gives an insight into the rabies situation in eastern Europe and Middle East. The paper deals with the outcome of a meeting of these countries which have recently joined hands in forming a Middle East and Eastern Europe Rabies Expert Bureau (MEEREB). It highlights the continued presence of canine rabies particularly in Egypt, Turkey, Iran, and Ukraine where several human deaths have occurred. The number of postexposure treatments is also on the increase in these countries Iran alone is administering more than 200,000 PEPs to humans bitten by dogs. 

The third paper by J. M. Reynes et al. describes a collaborative study between Pasteur Institute, Madagascar and Pasteur Institute, Paris on the situation of rabies in humans and animals in the island country of Madagascar located in the Indian Ocean in the eastern coast of Africa. The study analyses the rabies situation between 2005 and 2010 and the incidence of canine rabies was 54% and 9 confirmed human deaths occurred. Interestingly, rabies was not reported in bat species though one bat species did had antibodies to Lyssavirus. 

Though intradermal rabies vaccination (IDRV) with modern cell culture rabies vaccines is now widely used in many developing countries, the minimum potency requirement for IDRV is not very well documented and some countries have opted for a higher antigenicity (>2.5 IU per intramuscular dose). In an interesting study reported here by D. Brown et al. from the UK, it is clear that 100% seroconversion with antibody titers equal to or greater than 0.5 IU/mL can be achieved if a minimum cumulative antigenic content administered over a period is at least 2 IU. However, the results are based on a very small number of subjects and a larger study of this kind may help us understand the kinetics of immune response to varying antigenic content and dosage schedule of IDRV. 

Another interesting observation with regard to IDRV has been reported by T. Kamoltham et al. from Thailand. Here, they have compared the anamnestic response to booster doses of vaccine in school children who had received 2 (day 0 and 28) or 3 doses (days 0, 7, and 28) of preexposure primary vaccination in the past. They have shown that 2 doses by themselves induced protective levels of antibodies and the anamnestic response to booster doses administered 1, 3, or 5 years later was more or less, the same both in 2-dose and 3-dose primary groups. This aspect needs further investigation, as it has some implication in the cost of preexposure vaccination in children in rabies endemic countries.

In recent years, more and more information has accumulated on the structure and functions of rabies phosphoprotein (P). It has been shown that it is involved in the induction of innate immunity by promoting interferon production. Some groups are targeting P for genetic alteration and creating effective live attenuated vaccines for animal use. In a very interesting study, A. Vos et al. from Germany have created a P gene deletion mutant of SAD B 19 strain of rabies virus that seems to have a potential for use as live attenuated virus for immunization of animals. Such studies are indeed required to produce live vaccines that are not only highly immunogenic but also safe for untargeted animal species, specially humans.

Over all, this special issue on rabies contains timely, informative, and thought-provoking papers that may be of interest not only to basic researchers but also to medical and veterinary professionals engaged in prevention of human rabies and control of canine rabies.

*S. N. Madhusudana* *Deborah Briggs* *Hervé Bourhy*

